# Use of a porcine dermal collagen implant for contaminated abdominal wall reconstruction in a 105-year-old woman: a case report and review of the literature

**DOI:** 10.1186/s13256-015-0569-9

**Published:** 2015-04-29

**Authors:** Idit Melnik, Dimitry Goldstein, Boris Yoffe

**Affiliations:** Department of General and Vascular Surgery, Barzilai Medical Center, Hahistadrout St 2, Ashkelon, 78278 Israel

**Keywords:** Abdominal wall reconstruction, Contaminated fields, Geriatrics, Permacol™, Porcine dermis

## Abstract

**Introduction:**

Repair of contaminated abdominal wall defect in a geriatric patient is a challenge for the surgeon. We present the case of the oldest patient (105-years old) to successfully undergo a single-stage repair of a contaminated abdominal wall defect with a Permacol™ implant.

**Case presentation:**

A 105-year-old Caucasian woman presented to our emergency room with a clinical and radiological diagnosis of small bowel obstruction due to prior operative adhesions. She underwent laparotomy with small bowel resection and primary closure of her abdomen. There was total eventration of her bowel through the suture line 9 days after surgery. She underwent a second laparotomy that revealed no signs of peritonitis or turbid fluid. Her abdomen was closed with a 15×10cm Permacol™ implant sutured sublay with prolene sutures. Her postoperative period was unremarkable. After a follow-up period of 3 years and 2 months, there was no sign of recurrent hernia or wound contamination.

**Conclusion:**

We suggest that Permacol™ mesh can be considered an efficient alternative to primary closure or synthetic mesh in geriatric patients with contaminated abdominal wall defects.

## Introduction

The reconstruction of an abdominal wall defect or the repair of a large ventral hernia is a challenge for surgeons. In the repair of clean ventral hernia, the use of synthetic mesh can lead to a 50% reduction in hernia recurrence [1]. However, the use of synthetic mesh in cases of contamination is associated with serious complications such as fistula formation, adhesions, skin erosion and increased susceptibility to infections [2]. Thus, their use in contaminated fields is associated with high rates of morbidity and is strongly discouraged [3,4]. As a result, some advocate dividing definitive hernia repair into a two-stage approach [5,6]. The first operation entails removal of the infectious source, and then the patient must wait for 6 to 12 months before the second, definitive repair operation. For geriatric patients, however, this multistage method is especially problematic as most of them have numerous comorbidities. An operative protocol comprising two stages separated by an interval of at least 6 months aggravates the risks to geriatric patients for increased postoperative complications. Recently, the treatment focus has shifted to bioprosthetic meshes that provide strength and that promote host tissue incorporation and infection resistance, which together make them especially suited to treatment in contaminated fields when using a single-stage approach.

Permacol™ contains non-reconstructed porcine dermal collagen that has been stabilized against degradation by collagenase while retaining its elastin content and flexibility [7]. Recent case series have reported that Permacol™ is feasible and safe with the potential to be an acceptable alternative to prosthetic mesh in the repair of complicated and contaminated abdominal wall defects [4,8]. We present here our experience using Permacol™ mesh in the reconstruction of the abdominal wall of a 105-year-old patient with a 3-year follow up. To the best of our knowledge, this case is the oldest documented patient to undergo abdominal wall reconstruction with Permacol™ mesh.

## Case presentation

A 105-year-old Caucasian woman was admitted to our department complaining of diffuse abdominal pain with recurrent vomiting for a day. Her past surgical history included the repair of an incarcerated umbilical hernia 3 weeks prior and an open cholecystectomy 13 years before. Her past medical history included ischemic heart disease, atrial fibrillation with a cardiac pacemaker and hypertension. Despite her age, she was clear-minded, and she managed to fulfill her daily activities in her own home with the help of a nursing assistant. On physical examination her abdomen was distended and tympanic with tenderness in the postoperative scar near her umbilicus. A computed tomography of her abdomen demonstrated small bowel obstruction. A midline laparotomy through the previous scar revealed numerous adhesions of the small bowel. During adhesiolysis, one small bowel loop was opened and a resection with primary anastomosis was created. Her abdominal fascia was closed with polydioxanone loop and her skin was closed with tension nylon sutures.

There was a total eventration of her bowel through the suture line 9 days after surgery. She underwent a second laparotomy that revealed necrosis in the fascia edges. No signs of peritonitis or turbid fluid in peritoneal lavage were observed. Due to the fascial necrosis the field was believed to be contaminated. As a result, fascial edges were resected and instead of using a regular synthetic mesh, we decided to close her abdomen with a 15×10cm Permacol™ implant (Covidien) sutured sublay with prolene sutures. Her abdominal skin was closed with several single nylon sutures, leaving an interval of a few centimeters between one suture and the next, due to contamination. After the second surgery, the postoperative period was devoid of complications. As part of the conservative treatment approach, she was gradually returned to oral nutrition that was accompanied by normal bowel movements. Her abdominal skin was closed with close nylon sutures 7 days after the second surgery, and she reported feeling well, with no fever. Her abdomen was soft and non-tender without any signs of surgical site infection. Discharged later that day, she returned to her house as clear-minded and functional as she was prior to her admission to hospital.

During a follow-up period of 3 years and 2 months, there was no sign of either recurrent hernia or wound contamination. The patient reported that she felt good, and she had no complaints of abdominal pain. She died, 3.5 years after surgery, at the age of 108 of cardiac arrest unrelated to the abdominal surgery. For clarification, since the report is retrospective and obviously we cannot call the patient for a follow-up examination, all the data for the follow-up was collected from medical records, as the patient was hospitalized several times due to other reasons, not related to the surgery, and underwent physical examination by a doctor.

## Discussion

Permacol™ is a porcine-derived acellular dermal sheet composed predominantly of type I collagen (93 to 95%). During the manufacturing process the cellular components are removed and the collagen of the dermis is treated with hexamethylene diisocyanate (HMDI) to increase the degree of cross-linking [9]. The HMDI treatment also promotes neovascularization and tissue ingrowth, thereby creating an environment that favors antibiotic permeation [10,11]. In addition, due to its smooth surface and a lack of foreign body reaction when implanted, Permacol™ mesh can be placed equally well in contact with the bowel and with adipose tissue.

The use of Permacol™ in hernia repair was first proposed in 1984 [12]. Subsequent *in vivo* studies evaluating the different biologic meshes reported that Permacol™ is a safe prosthetic material for ventral hernia repair, supporting hernia healing by strengthening the damaged tissue [13,14]. Catena *et al.* [8] reported a case series of seven patients, age range of 69 to 83, who were treated with Permacol™ for complicated incisional hernias. In a mean follow-up of 11.1 months no recurrence or wound infection was observed. Another study [15] reported a case series of 28 patients ranging in age from 44 to 83 with acute and chronic abdominal wall defects, environments with potentially contaminated wounds, treated with Permacol™ mesh. They described three recurrences (15%) in a median follow-up of 16 months. Likewise, Loganathan *et al.* [7] reported the cases of 15 patients, age range 36 to 76, who underwent repair of complex or recurrent hernia with Permacol™, some of the patients had infected surgical fields. Two recurrences were reported, one in a parastomal hernia within 30 days, most likely due to surgical technique, and the other in an incisional hernia. Although not clearly stated in the paper, the latter case was probably a late recurrence.

In spite of such promising results, the use of Permacol™ in contaminated surgical fields is still controversial. In fact, recent reports have cast doubt on those results. García-Pumarino *et al.* [16] reported a comparative *in vitro* and *in vivo* study of collagen meshes (Collamend®, Surgisis® and Permacol™®) to the polytetrafluoroethylene mesh, Preclude®, in contaminated surgical fields. They reported that the collagen bioprostheses failed to show any bacterial adhesion or bacterial clearance benefits. In addition, Rosen *et al.* [17] reported a large study of 128 patients, mean age 58.2±13.5 years, evaluating the long-term results after repairing contaminated abdominal wall defects with biologic meshes. Although the biologic meshes that were tested (Strattice^™^, AlloDerm^®^, Biodesign, XenMatrix^™^ and BioA) did not include Permacol™, and in our case the use of Permacol™ with a follow up of approximately 3 years revealed no signs of hernia recurrence, this finding still raises the question of the long-term efficacy of Permacol™. For clarification, recently studies report favorable outcomes with synthetic mesh in a contaminated ventral hernia [18]. However, their usage in a contaminated field is not yet a standard of care, and due to this controversial issue the use of a biological mesh is still a reasonable and effective technique.

While the general issue of abdominal wall repair in the presence of contamination is controversial, it becomes even more complicated for geriatric patients, who often have multiple comorbidities. This situation precludes the use of a two-stage surgical procedure with geriatric patients, in whom the potential increase in operative risk is unacceptable. Moreover, the high rates of pulmonary diseases in these patients rule out the use of a direct suture, which will increase intra-abdominal pressure, thereby influencing respiratory dynamics [19].

Based on the literature reviewed here, we decided in our case of a 105-year-old patient with bowel eventration to repair her abdominal wall defect with Permacol™ mesh. Over the course of a follow-up period of 3 years and 2 months, we did not observe any sign of recurrent hernia or long-term infectious complications. In addition to the surgical technique of using a biological mesh, we must mention that it was a combination of better perioperative care and preoperative preparation (especially in a geriatric patient) that were the basis of the successful treatment.

## Conclusions

The repair of a contaminated abdominal wall defect in a geriatric patient is a challenge for the surgeon. Due to their high cost and the fear of postoperative complications, many surgeons decide not to use a biological mesh in a geriatric patient. We present the case of the oldest, to the best of our knowledge, patient (105-years old) to have successfully undergone a single-stage repair using a Permacol™ implant. We suggest that Permacol™ mesh can be considered an efficient alternative to primary closure or synthetic mesh in geriatric patients with contaminated abdominal wall defects.

## Consent

Written informed consent was obtained from the patient’s next-of-kin for publication of this case report and any accompanying images. A copy of the written consent is available for review by the Editor-in-Chief of this journal.
